# Supporting People with Intellectual Disability and Their Carers to Understand the Risk of Constipation with Clozapine Therapy Utilising a Brief Educational Tool. A Quality Improvement Project

**DOI:** 10.1192/bjo.2025.10457

**Published:** 2025-06-20

**Authors:** Daniel Agius, Catherine Walton, Rhiannon Lewis, Abdellatif Elkhashab, Matthew Jones

**Affiliations:** 1 Cardiff and Vale University Health Board, Cardiff, United Kingdom; 2 Swansea Bay University Health Board, Cardiff, United Kingdom; 3 Swansea Bay University Health Board, Swansea, United Kingdom; 4 Swansea Bay University Health Board, Rhondda Cynon Taf, United Kingdom

## Abstract

**Aims:** People with Intellectual Disabilities (PwID) have, on average, a life expectancy 20 years less than that of the general population. The Learning Disabilities Mortality Review found that in 23% of deaths among PwID, constipation was a long-term health problem. In the past year, Swansea Bay University Health Board’s (SBUHB) Mental Health and Learning Disability Delivery Unit reported four incidents of constipation among PwID living in the community, with one fatality.

Patients prescribed clozapine are more vulnerable to constipation due to side effects. This Quality Improvement (QI) project aimed to assess the current knowledge about constipation among PwID prescribed clozapine, along with their carers, and to use a brief educational tool to address knowledge gaps.

**Methods:** Stakeholder analysis, fishbone diagram, and process mapping were undertaken to create a driver diagram and identify change ideas. Education was chosen as the primary driver for this project, with a focus on assessing understanding, and providing patient and carer education. The project targeted all PwID prescribed clozapine within three geographical areas: Cardiff, Swansea, and Rhondda Cynon Taf. An initial knowledge survey was administered to both patients and carers, followed by a face-to-face educational session using an Easyread leaflet. Knowledge was reassessed one week later.

**Results:** Seven educational sessions were held, with patients and their primary carers participating. The knowledge survey revealed that all patients understood the basic concept of constipation, but fewer understood its health risks (30%) and the recommended frequency of bowel movements (14%). Knowledge improved and was retained one week after the education session, with 60% understanding the health risks and 71% knowing the recommended frequency of bowel movements. Carers demonstrated improved knowledge, particularly in using the Bristol Stool Chart. All carers recognized the increased risk of constipation among PwID and its potential fatal consequences.

**Conclusion:** This project demonstrated that targeted, brief educational interventions can effectively improve the knowledge of PwID and their carers regarding the risks of constipation associated with clozapine therapy. The results emphasise the importance of accessible information and suggest that continuing education is necessary for both PwID and carers. It also highlighted the importance of a stable and educated carer workforce, with appropriate training at induction. The future aim of the project team is to develop an online educational programme for carers about constipation, and how to seek timely and effective support.

